# Supplementary material for: Computer algorithms for automated detection and analysis of local Ca2+ releases in spontaneously beating cardiac pacemaker cells
Source: PLoS One. 2017 Jul 6;12(7):e0179419. doi: 10.1371/journal.pone.0179419 (PMC5500000; doi:10.1371/journal.pone.0179419)
Supplement: S1 Table — Results are mean values obtained in one pacemaker cycle in each cell. Grey area shows specific input program parameters to analyze recordings in each cell. (PDF) [file pone.0179419.s001.pdf]

S1 Table

Results of LCR detection analysis performed by XYTEventDetector using Ca signal recordings by PCO.edge 4.2 CMOS camera in SA node cells of rabbit and guinea pig, 4 cells of each species.

Parameters	Rabbit					Guinea Pig				
	**cell#1**	**cell#2**	**cell#3**	**cell#4**	**Mean**	**cell#1**	**cell#2**	**cell#3**	**cell#4**	**Mean**
Cycle Length (ms)	540	690	480	560	567.5	1370	690	380	800	810.0
Path Size (μm^2)	2.427	5.211	3.01994	1.216	3.0	5.641	1.218	7.233	1.03	3.8
Duration (ms)	12.5	14.3	12.3	11.2	12.6	24.4	10.8	15.5	11.1	15.5
LCR Period (ms)	471.5	446.7	221.1	370.6	377.5	843	451.4	229.8	474.7	499.7
LCRs/Cycle	59	48	81	119	76.8	184	87	52	75	99.5
False positives rate%	0	0	6.7	0.97	1.9	0	0	0	0	0.0
Max Filter	100	100	75	100	93.8	100	100	100	100	100.0
SD Detection	0.4	0.4	0.4	0.4	0.4	0.4	0.3	0.3	0.3	0.3
SD Termination	1.5	1.5	1.5	1.5	1.5	1.5	1.5	1.5	1.5	1.5
Search Distance	8	8	8	4	7.0	8	7	8	7	7.5
Size Threshold	7	7	7	7	7.0	7	7	7	7	7.0
Intensity Threshold	50	50	50	50	50.0	50	50	50	50	50.0
% Transient Cutoff	99	70	90	90	87.3	70	70	70	70	70.0
Cell size (μm2)	683.13	281.73	654.91	415.91	508.9	483.28	925.5	1093.06	705.81	801.9

Results are mean values obtained in one pacemaker cycle in each cell. Grey area shows specific input program parameters to analyze recordings in each cell.
