# Supplementary material for: Computer algorithms for automated detection and analysis of local Ca2+ releases in spontaneously beating cardiac pacemaker cells
Source: PLoS One. 2017 Jul 6;12(7):e0179419. doi: 10.1371/journal.pone.0179419 (PMC5500000; doi:10.1371/journal.pone.0179419)
Supplement: S2 Table — Results are mean values obtained in 3 pacemaker cycles in each cell. N/D, not determined. Grey area shows specific input program parameters to analyze recordings in each cell. (PDF) [file pone.0179419.s002.pdf]

S2 Table

Results of LCR detection analysis performed by XYTEventDetector using Ca signal recordings by Hamamatsu C9100-12 CCD camera in SA node cells of rabbit and guinea pig, 4 cells of each species.

**Parameters**	Rabbit					Guinea Pig				
	**cell#1**	**cell#2**	**cell#3**	**cell#4**	**Mean**	**cell#1**	**cell#2**	**cell#3**	**cell#4**	**Mean**
Cycle Length (ms)	536	480	361	512	472.25	577	515	457	411	490
Path Size (µm^2)	9.56	8.96	10.44	6.75	8.92	9.88	12.43	11.38	10.18	11.0
Duration (ms)	14.29	13.2	14.32	14.18	14.0	13.45	16.33	15.75	13.52	14.76
LCR Period (ms)	230.27	209.85	126.97	193.1	190.0	369.57	269.18	260.09	257.32	289.04
LCRs/Cycle	61	66	44	98	67.25	86	53	38	47	56
False positives rate%	N/D	N/D	N/D	N/D		N/D	N/D	N/D	N/D	
Max Filter	80	80	170	150		100	150	100	100	112.5
SD Detection	0.4	0.5	0.5	0.5		0.3	0.4	0.4	0.4	0.375
SD Termination	1	2	1	1		1	1	1	1	1
Search Distance	3	3	3	3		4	4	4	4	4
Size Threshold	5	5	5	5		5	5	5	5	5
Intensity Threshold	50	50	50	50		50	50	50	50	50
% Transient Cutoff	99	98	99	99		99	99	99	99	
Cell size (µm2)	N/D	N/D	N/D	N/D		N/D	N/D	N/D	N/D	99

Results are mean values obtained in 3 pacemaker cycles in each cell. N/D, not determined. Grey area shows specific input program parameters to analyze recordings in each cell.
